# Hitting the Optimal Vaccination Percentage and the Risks of Error: Why to Miss Right

**DOI:** 10.1371/journal.pone.0156737

**Published:** 2016-06-22

**Authors:** Michael J. Harvey, Lisa A. Prosser, Mark L. Messonnier, David W. Hutton

**Affiliations:** 1 Department of Health Management and Policy, School of Public Health, University of Michigan, Ann Arbor, Michigan, United States of America; 2 Child Health Evaluation and Research Unit, Division of General Pediatrics, University of Michigan Medical School, Ann Arbor, Michigan, United States of America; 3 National Center for Immunization and Respiratory Diseases, Centers for Disease Control and Prevention, Atlanta, Georgia, United States of America; University British Columbia, CANADA

## Abstract

**Objective:**

To determine the optimal level of vaccination coverage defined as the level that minimizes total costs and explore how economic results change with marginal changes to this level of coverage.

**Methods:**

A susceptible-infected-recovered-vaccinated model designed to represent theoretical infectious diseases was created to simulate disease spread. Parameter inputs were defined to include ranges that could represent a variety of possible vaccine-preventable conditions. Costs included vaccine costs and disease costs. Health benefits were quantified as monetized quality adjusted life years lost from disease. Primary outcomes were the number of infected people and the total costs of vaccination. Optimization methods were used to determine population vaccination coverage that achieved a minimum cost given disease and vaccine characteristics. Sensitivity analyses explored the effects of changes in reproductive rates, costs and vaccine efficacies on primary outcomes. Further analysis examined the additional cost incurred if the optimal coverage levels were not achieved.

**Results:**

Results indicate that the relationship between vaccine and disease cost is the main driver of the optimal vaccination level. Under a wide range of assumptions, vaccination beyond the optimal level is less expensive compared to vaccination below the optimal level. This observation did not hold when the cost of the vaccine cost becomes approximately equal to the cost of disease.

**Discussion and Conclusion:**

These results suggest that vaccination below the optimal level of coverage is more costly than vaccinating beyond the optimal level. This work helps provide information for assessing the impact of changes in vaccination coverage at a societal level.

## Introduction

Vaccines provide a benefit to society by preventing disease and controlling the spread of disease in the population [1]. However, vaccination programs also require large investments [2]. As costs of newer vaccines increase, understanding and determining the relative value of various vaccination programs is becoming increasingly important [3]. Because some vaccination programs may be competing for the same set of resources, funding one vaccine could come at the expense of another. Vaccinating too few individuals risks higher costs of disease whereas vaccinating too many risks spending too much on things like education, targeting, administration, and vaccine doses. Given the competing interests of societal benefit and high cost of implementation, it is important to identify the optimal level of vaccination coverage for a disease in order to avoid the costs of over- or under-vaccination and to help prioritize efforts to increase vaccination coverage [2, 4–6].

There are two common approaches to optimizing vaccination coverage levels. The first is to determine the level of vaccination coverage required to reduce the reproductive rate of the disease (R_0_) to 1. Reducing the R_0_ to 1, achieves indirect effects and stops the spread of disease in a population [5, 7–9]. The other approach is to minimize the total costs of vaccination [4, 6] by varying levels of vaccination coverage. The objective of this research was to examine the optimal population vaccination coverage levels for a variety of disease and vaccine characteristics to investigate the effect on costs given sub-optimal vaccination coverage.

## Methods

The classic epidemic Susceptible–Infected–Recovered (SIR) model was modified with vaccination included as a fourth model state [10]. For simplicity, all diseases were assumed to be non-fatal, and vaccination was assumed to provide permanent protection against disease. The model is described by the following series of differential Eqs 1–4.

dS/dt=−λS (1)

dI/dt=λS−γI (2)

dR/dt=γI (3)

dV/dt=0 (4)

Eqs 1–4. Equation set for SIRV model: Notes: S: Susceptible. I: Infected. R: Recovered. V: Vaccinated. λ: Force of Infection = (βI/N). β: Transmission Rate from Susceptible to Infected = (γ*R_0_). γ: Recovery Rate = (1 / infection period (days)). R_0_: Reproductive Rate. N: Total population size.

General parameters that could be representative of a wide range of vaccine-preventable conditions were used; Table 1 provides a list of parameter inputs for the SIRV model.

**Table 1:** Model inputs and ranges for SIRV model.

Disease and vaccination parameters	Values
**Total population size, N**	1000001
**Reproductive Rate (R_0_)**	1.1–10.0
**Infection period (days)**	7, 14, 21, 42
**Recovery Rate (γ)**	(0.02–0.14)
**Transmission Rate from Susceptible to Infected (β)**	(0.03–1.4)
**Vaccine efficacy (%)**	80–100

The model was seeded with one infected individual and a population of 1,000,000 that were split between susceptible and effectively vaccinated depending on the vaccine coverage and efficacy. Vaccination coverage was varied from 0 to 100% to provide data for determining the population vaccination coverage to achieve the minimum total cost; hereafter the optimal vaccination level. Vaccine efficacy is defined as the fraction of individuals being fully-protected by the vaccine. Those not fully-protected were assumed to be completely susceptible. Each model was run over a two-year time horizon. Two-years was selected as it provided enough time to complete one full outbreak of the disease for each R_0_ and infection period tested. The model was constructed using the statistical software R (version 3.2.3). The model predicted the following set of outcomes for each run: the remaining number of people susceptible to disease and the number of people either still infected or recovered from disease after the two-year time horizon.

### Optimal Vaccination Level Analysis

SIRV model outcomes were utilized to determine the optimal coverage and provide insights into the value of incremental changes to coverage. The number of people vaccinated, along with the number of people either still infected or recovered from disease were used to explore the relationship between the cost of disease and cost of vaccination and how this relationship alters the optimal vaccination level. The main outcomes of interest in all analyses were the total cost and the associated optimal vaccination level.

Eq 5 shows total cost (TC) for any level of vaccination coverage. TC was constructed using results of the SIRV model, parameters for disease costs and a disease to vaccine cost ratio (DVCR). The cost of disease combines three components: 1) Dollar cost of disease; 2) Quality adjusted life years (QALYs) lost due to disease; 3) Willingness-to-pay (WTP) per QALY. To examine a variety of possibilities for the utility lost due to disease, a range of possible QALY losses were selected. As there is no currently accepted threshold for a WTP per QALY in the US [11], several different WTP values were tested to encompass the plausible ranges for the societal WTP for QALYs lost. QALYs were monetized by multiplying the WTP per QALY and the number of QALYs lost due to disease, as shown in Eq 5. The list of QALYs lost, and WTP per QALY are provided in Table 2. Each component of the cost of disease was examined in one-way sensitivity analysis to determine its effect on the optimal vaccine coverage.

**Table 2:** Model inputs for optimal vaccination level analysis.

**Variable**	**Base-case**	**Other values**
Disease cost per person ($)	1000	10, 100, 2000, 2500, 5000, 10000
QALYs lost per person	0.005	0.0001, 0.001, 0.01, 0.025, 0.05, 0.1, 0.5, 1
Willingness to pay per QALY ($)	100000	25000–250000, by 25000
Disease to vaccine cost ratio (DVCR)	15	1, 2, 3, 4, 5, 10,15, 20, 25, 50, 75, 100, 1000, 10000

$${TC}_{x}=(x\times N_{Sus}\times {Cost}_{Vac})+\left[(N_{Rec}+N_{Inf})\times ({Cost}_{Dis}+(QALYs\;Lost\times \frac{WTP}{QALY}))\right]$$ (5)

Eq 5. Total cost: *N*_*Sus*_: Initial number of susceptible people. *N*_*Rec*_: Number recovered from infection after simulated outbreak epidemic (2 years). *N*_*Inf*_:: Remaining number infected after simulated outbreak (2 years). *x*: Level of vaccination coverage (%). *Cost*_*Dis*_: Disease cost ($). *Cost*_*Vac*_: Cost of vaccine ($). Subject to disease dynamics Eqs 1–4.

Once the parameters for disease cost were selected, disease cost was assumed to be fixed for each analysis. The cost of the vaccine was determined by altering the DVCR. As disease cost was fixed, it is important to note that altering this ratio is indicative of change in the vaccine cost not the disease cost. An increase in DVCR thus implies a reduction in the cost of the vaccine. The range of the DVCR is provided in Table 2.

Eq 5 demonstrates there is a total cost (*TC*_*x*_) for each level of population vaccination coverage (*x*). The optimal vaccination level is defined as the minimum possible total cost when vaccination coverage is varied (*TC*_x*_)

$${TC}_{x^{*}}=\begin{array}{c} min\\ x \end{array}{TC}_{x}$$ (6)

Eq 6. Optimal vaccination level: *x*: Level of vaccination coverage (%). *TC*: Total cost as defined in Eq 5. 0 ≤ *x* ≤ 100. Subject to disease dynamics Eqs 1–4.

### Total Costs of Over- and Under-Vaccination

To compare the cost of under-vaccinating (i.e., achieving a vaccination coverage lower than the optimal level) to over-vaccinating (i.e., achieving a vaccination coverage higher than the optimal level), we created a metric of the excess cost above the minimum, defined by Eq 7. The symmetry or asymmetry in the costs of vaccinating at levels less than and greater than the optimal level was examined.

$$y(x)={TC}_{x}-{TC}_{x^{*}}$$ (7)

Eq 7. Excess cost above minimum: *y(x)*: Difference from the minimum total cost at vaccination percentage *x*. *TC*_*x*:_ Total cost as defined in Eq 5. *TC*_x*_: Minimum total cost as defined in Eq 6. Subject to disease dynamics Eqs 1–4.

### Optimal Vaccination Level and Indirect Effects

The economically optimal vaccination coverage level, *x*, as defined by minimizing *TC* may be different from the epidemiologic minimum level of vaccination coverage, *z*, where indirect effects lead to a decline in the incidence of infection (“herd immunity threshold”)(shown by Eq 8). To study the relationship between these two values of vaccination coverage, we used a bisection search algorithm to determine the approximate DVCR at which the economically-optimal vaccination coverage level, *x*, is equivalent to the indirect effects threshold, *z*, for various R_0_s between 1.1 and 10.0.

$$z=\left(1-\frac{1}{R_{0}}\right)\frac{1}{vaccine\;effectiveness}$$ (8)

Eq 8. Indirect Effects Threshold: *z*: Level of vaccination coverage (%). R_0_: Reproductive Rate

## Results

**Fig 1:**
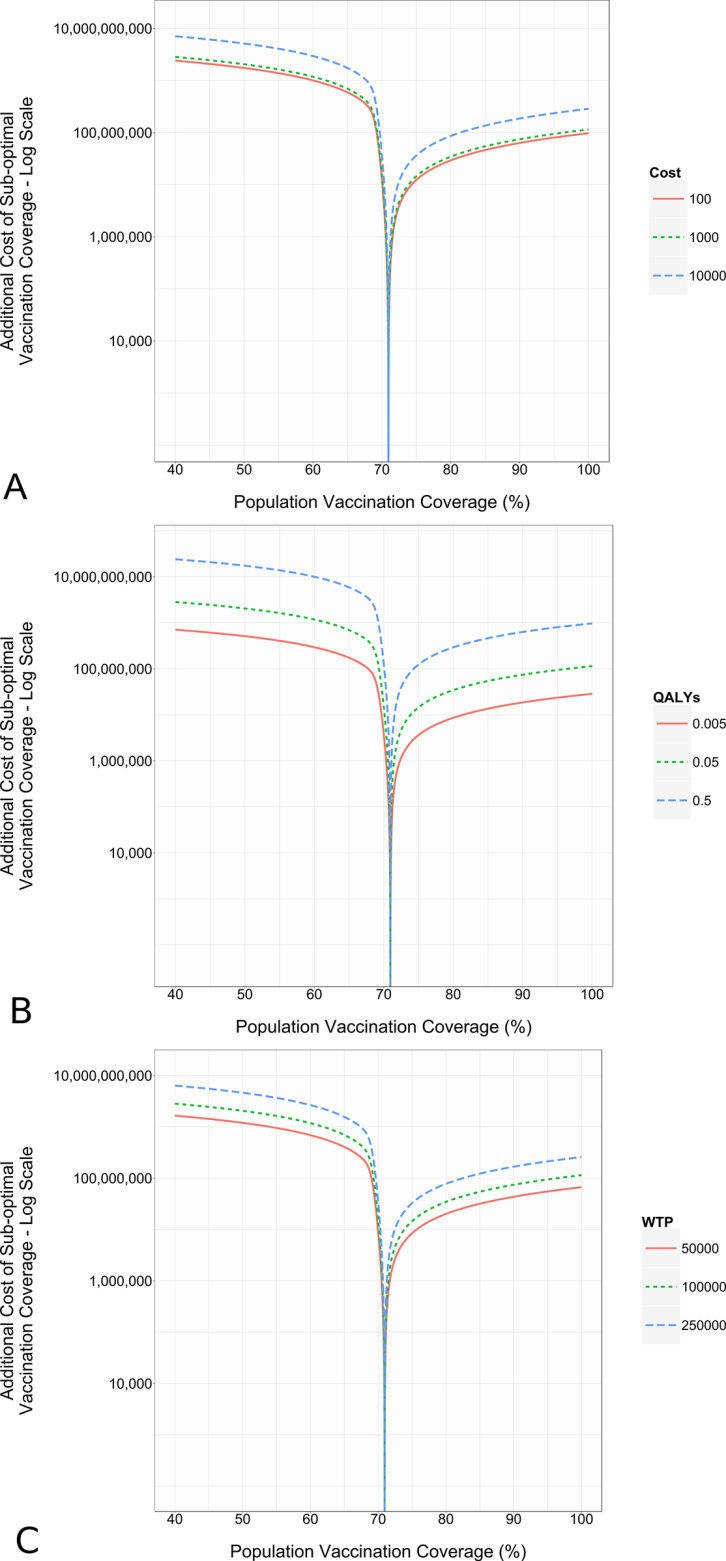
Sensitivity analysis results. R_0_: 3.5, DVCR: 15. Values for parameters that remained constant during one-way analysis: Direct costs: $10000. QALYs Lost: 0.05. WTP: $100000. (A) One-way sensitivity analysis direct costs of disease. (B) One-way sensitivity analysis on QALYs lost due to disease. (C) One-way sensitivity analysis on number WTP per QALY.

Sensitivity analysis reveals that the optimal coverage level is robust to changes in the individual components of disease cost. Fig 1 provides an example of the result from the one-way sensitivity analysis where the components of disease cost are presented at different values and DVCR is held constant. Fig 1 shows the additional costs of sub-optimal vaccination coverage at a population level. The minimum point on Fig 1 indicates the optimal vaccination coverage (*TC*_x*_) for this combination of disease and vaccine characteristics. Results show that the optimal vaccination coverage does not change when the individual components of disease cost are changed. What does change is the additional cost due to not achieving the optimal coverage level. That is, from an optimization perspective, the optimal coverage level is not sensitive to components of the cost of disease or the cost of the vaccine. Rather, it is driven by the DVCR.

### Total Costs of Over- and Under-Vaccination

**Fig 2:**
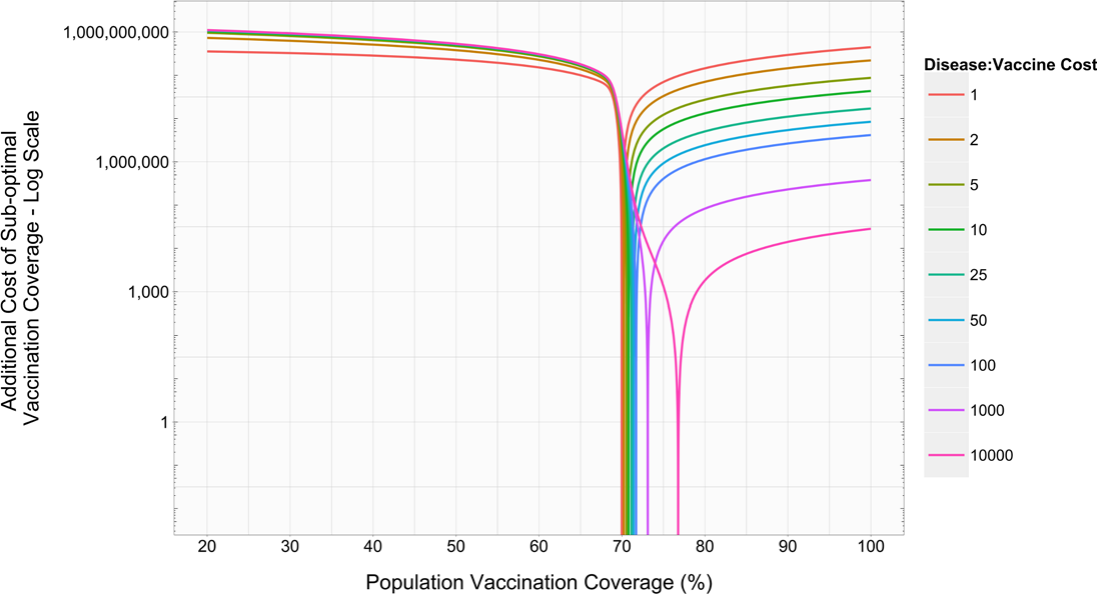
Costs of vaccinating short of and beyond optimal levels. R_0_: 3.5. Vaccine Efficacy: 100%. Total Disease Cost: $1,500.

Fig 2 provides an example of the symmetry of excess costs near optimal coverage levels. Results indicate that small deviations from optimal coverage can greatly impact additional costs. Not surprisingly, as the cost of a vaccine drops (DVCR increases), the optimal level of population vaccination coverage increases. Fig 2 shows that in this example; as DVCR varies over several orders of magnitude, the optimal vaccination coverage (the horizontal position where the graph line is minimized) may vary by a few percentage points. Under nearly all conditions, missing the optimal coverage level by vaccinating a larger fraction of the population will result in lower excess costs than missing the optimal coverage level by vaccinating a smaller fraction of the population. The only instance when vaccination beyond the optimal coverage level can be more expensive is when the disease and vaccine have the same cost (i.e., DVCR = 1.0).

### Optimal Vaccination Level and Indirect Effects

**Fig 3:**
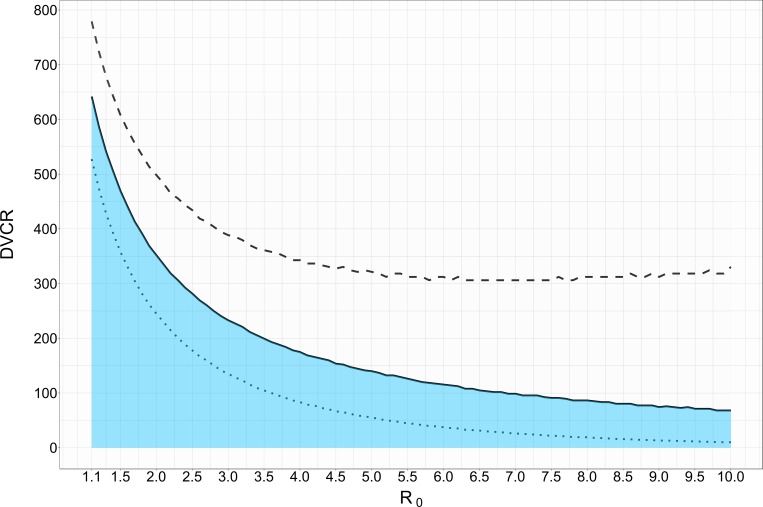
DVCR and the relationship between the economically-optimal population coverage level and the indirect effects threshold. γ = 14 days. Solid line: Approximate DVCR where the economically-optimal vaccination level equals indirect effects threshold for a particular R_0_. Dashed line: Economically-optimal coverage levels + 0.5 percentage points from indirect effects threshold. Dotted line: Economically-optimal coverage levels– 0.5 percentage points from the indirect effects threshold.

Fig 3 shows how the economically-optimal vaccination percentage, *x*, compares to the level of vaccination coverage, *z*, required to achieve indirect effects. The solid line in Fig 3 indicates the DVCR at which the economically-optimal vaccination coverage, *x*, as defined by $TC_{x^{*}}$, is approximately equivalent to the population coverage, *z*, needed to achieve the indirect effects threshold (*z* ± 0.01 (percentage points), for a particular R_0_. Results show that given disease and vaccine characteristics and costs, there may be scenarios when the economically-optimal vaccination percentage, *x*, is less or greater than the indirect effect threshold, *z*. Fig 3 indicates that if the DVCR is sufficiently high and the disease is highly transmissible, it may be optimal to vaccinate beyond the point of indirect effects (i.e., the optimal may be above the black line). Conversely, if the DVCR is low (vaccine is expensive relative to disease) and the disease is not highly transmissible, it may be economically-optimal to vaccinate below the indirect effects threshold (i.e., the optimal may be below the black line).

### Policy Example

**Fig 4:**
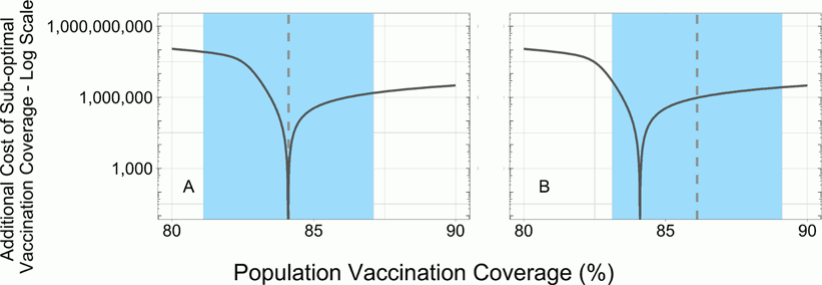
Policy example. R_0_: 5.0. Vaccine Efficacy: 95%. DVCR: 25. Disease Cost $1500. Dotted Line: Target coverage level. Shaded region: ± 0–2 percentage point from target coverage level. (A) Target coverage at optimal coverage level. (B) Target coverage level two percentage points above the optimal.

One example of a policy recommendation generated from these results could be setting the target vaccination coverage level as little as two percentage points above the optimal level to help minimize total expenditures. Fig 4 provides a visualization of this policy. If a vaccination target is set at the optimal level and not achieved exactly, excess costs will be incurred. If the vaccination target is set at the optimal level and missed on the under-vaccination side by a small margin excess costs could be extremely high (e.g., ~ $100,000,000 for a population of 1 million people). Conversely, if the target coverage level is set above the optimal and missed by the same margin costs are much less burdensome (e.g., < $10,000,000 for a population of 1 million people).

## Discussion

The objective of this research was to examine the optimal population vaccination coverage levels by identifying the minimum total costs for a variety of disease and vaccine characteristics, and to use this information to gain insights to the costs and benefits as vaccination coverage changes incrementally. Under reasonable conditions (i.e., the vaccine being less expensive than the disease it protects against), results indicate there are much higher costs to vaccinating at levels less than the optimal coverage level compared to vaccinating at levels greater than the optimal. Although several papers provide examples for coverage optimization using similar or other model structures [2, 4, 5, 12, 13], this paper adds to the optimization coverage literature in some important ways. First, to our knowledge, this is the first paper that examines the change in symmetry in over- and under-vaccination near optimal levels of coverage. A key insight from this paper is the additional margin that exists by exceeding the optimal coverage level and the possible policy implications. To some extent, the idea of additional margin beyond optimal coverage is intuitive, however the rapid rate of change in additional expenditures is difficult to capture without visualization. Also, understanding changes in symmetry for different vaccines could aid priority setting for promotion of increased coverage for different vaccines.

Mass vaccination is complicated and requires many processes to work simultaneously, and many things can prevent a target coverage level from being obtained. For example, people may cancel appointments or not show; vaccines may expire before being used; or people may opt out of vaccination for religious, political, or social reasons. These and other reasons would contribute to target coverage not being achieved; with coverage rates likely falling left of (lower than) a set target. Contrary, for a vaccination target to be surpassed, many processes would need to work better than planned. Therefore, in examining policy recommendations such as the example from Fig 4, a strategy that employs vaccination beyond the optimal level as the target may be useful for minimizing costs as missing on the left side of the target could even lower net costs, especially if the vaccine is inexpensive compared to disease.

If the indirect effects threshold is low, such is the case when the R_0_ is small, additional complications may arise from policy considerations that would suggest that it is optimal to vaccinate near this threshold.

The methods used to find the optimal as well as providing a graphical example of the symmetry could be useful for priority setting for different vaccines. For example, if the optimal coverage level for vaccine A is 90% and actual population coverage is at 95%, while the optimal coverage for vaccine B is 80% but actual coverage of vaccine B is 60%, this might suggest that resources be reallocated from promoting vaccine A to promoting vaccine B. How a regulatory body could effectively communicate and equitably implement a policy is beyond the scope of this paper; we do acknowledge that using only results from this type of model may provide extra additional difficulties for decision makers. However, this model and its methods are useful in situations where full coverage has not been achieved or where prioritization may be required. Regardless, it is important to acknowledge that model results like this are only one type of evidence considered when making vaccination policy or recommendations.

Because the aim of this study was to explore the potential impact of changes in coverage for application to a wide range of vaccine-preventable conditions, the classic epidemic model was chosen as it can reflect a range of disease conditions being considered. The classic epidemic model illustrates how diseases progress through a population, easily allows us to track the course of a hypothetical disease, and can provide insights for a wide variety of diseases as most infectious diseases follow some variation of the susceptible-infected-recovered process. Although we chose this simplified model instead of a more complicated disease-specific model (with additional details that may have obscured the key insights), we see these methods as something that could be readily adapted for dynamic transmission models of specific diseases.

There are some limitations with these models. First, our model assumes homogeneous population mixing. While assumption holds for some diseases, it would be important to account for population heterogeneity for other diseases. Second, there is no age structure in these models. Accounting for the age structure of the population can help better account for associated costs and vaccination coverage needs. Models with age structure could also evaluate the benefits of expanding childhood vaccination for vaccines with lifelong protection. However, these results could account for age groups if one assumes that the models are applicable to only a single group of interest. Therefore, instead of the model representing people of all ages, the model would represent a population where every person is a child or an adult. Third, assumptions for vaccination and disease outcomes vary by condition. For example, people both vaccinated and unvaccinated lose immunity over time for some diseases. Accounting for vaccination immunity and natural immunity loss could play an important role in the total cost of vaccination. Fourth, models were closed cohort. As the population changes (i.e., people being born, and people dying) those entering the model would need to be vaccinated to keep coverage constant. Although including these extra details of the models would provide extra accuracy for different diseases, accounting for all of these additional pieces, would make the model structure much more complex and would make it less generalizable. This paper also serves as a possible method that could be readily applied to existing dynamic transmission models. Examining more complex models and previously constructed dynamic transmission models using these methods is an area for continuing research.

Costs are a possible limitation of this paper. The cost of the vaccine is a combination of the vaccine itself as well as the administration cost; we acknowledge that administration costs are likely to be dynamic given the vaccine and the level of coverage. Additionally, considering the impact of economies of scale could be important for events like childhood vaccination (where many vaccines are received at one time) or the impact when multiple doses are required to complete a vaccination schedule. However, as we find that only parameter that impacts our results is DVCR, this ratio could certainty account for both vaccine and administration costs, as well as multiple doses, and economies of scale. A further consideration of cost would be the impact of symptomatic versus asymptotic transmission. In our model we assume that the cost of disease is the average cost of disease across a population which includes both asymptomatic and symptomatic transition. Therefore, the DVCR will change as the percentage of symptomatic versus asymptomatic cases changes. Additional work would be required for specific diseases to accurately calculate the total cost of vaccination, accounting for both direct and indirect costs, and the cost of disease, accounting for symptomatic or asymptomatic transmission.

## Conclusion

In summary, we have evaluated the optimal level of vaccination coverage considering the population costs and benefits. Results suggest that vaccination at levels below the optimal is costlier than vaccination at levels beyond the optimal when vaccination costs less than the disease. Equipping decision makers with the knowledge that it is possible to over-vaccinate beyond the optimal by small margins and only incur minimal additional costs is valuable. Further research could examine more complex models, specific vaccines, and specific diseases to determine how generalizable these results are. If results prove to be generalizable they could be used to help inform practice and policy decisions **or** set target recommendations when mass vaccination may be required.
